# Brain-targeted delivery of neuroprotective survival gene minimizing hematopoietic cell contamination: implications for Parkinson’s disease treatment

**DOI:** 10.1186/s12967-023-04816-x

**Published:** 2024-01-13

**Authors:** Min Hak Lee, Sukyeong Kang, Ki-Hwan Um, Seok Won Lee, Hyorin Hwang, Kyunghwa Baek, Jin Woo Choi

**Affiliations:** 1 Department of Pharmacology, College of Pharmacy, Kyung Hee University, Seoul, 02447 Republic of Korea; 2 Department of Biological and Medicinal Science, Graduate School, Kyung Hee University, Seoul, 02447 Republic of Korea; 3 Department of Pharmacology, Institute of Regulatory Innovation Through Science, Kyung Hee University, Seoul, 02447 Republic of Korea; 4 Generoath Ltd., Seoul, 04168 Republic of Korea; 5 Department of Pharmacology, College of Dentistry and Research Institute of Oral Science, Gangneung-Wonju National University, Gangneung-si, Gangwon-do 25457 Republic of Korea

**Keywords:** AIMP2 splicing variants, DX2, miRNA, Parkinson’s disease, Adeno-associated virus, Gene therapy

## Abstract

**Background:**

Neurodegenerative diseases, including Parkinson's disease, Amyotropic Lateral Sclerosis (ALS) and Alzheimer's disease, present significant challenges for therapeutic development due to drug delivery restrictions and toxicity concerns. Prevailing strategies often employ adeno-associated viral (AAV) vectors to deliver neuroprotective survival genes directly into the central nervous system (CNS). However, these methods have been limited by triggering immunogenic responses and risk of tumorigenicity, resulting from overexpression of survival genes in peripheral blood mononuclear cells (PBMC), thereby increasing the risk of tumorigenicity in specific immune cells. Thus, by coding selectively suppressive microRNA (miRNA) target sequences in AAV genome, we designed CNS-targeted neuroprotective gene expression vector system without leakage to blood cells.

**Methods:**

To minimize the potential for transgene contamination in the blood, we designed a CNS-specific AAV system. Our system utilized a self-complementary AAV (scAAV), encoding a quadruple repeated target sequence of the hematopoietic cell-specific miR142-3p at the 3' untranslated region (UTR). As a representative therapeutic survival gene for Parkinson’s disease treatment, we integrated DX2, an antagonistic splice variant of the apoptotic gene AIMP2, known to be implicated in Parkinson's disease, into the vector.

**Results:**

This configuration ensured that transgene expression was stringently localized to the CNS, even if the vector found its way into the blood cells. A single injection of scAAV-DX2 demonstrated marked improvement in behavior and motor activity in animal models of Parkinson’s disease induced by either Rotenone or 1-methyl-4-phenyl-1,2,3,6-tetrahydropyridine (MPTP). Importantly, comprehensive preclinical data adhering to Good Laboratory Practice (GLP) standards revealed no adverse effects in the treated animals.

**Conclusions:**

Our CNS-specific vector system, which encodes a survival transgene DX2, signifies a promising avenue for safe gene therapy, avoiding unintended expression of survival gene in blood cells, applicable to various neurodegenerative diseases.

**Supplementary Information:**

The online version contains supplementary material available at 10.1186/s12967-023-04816-x.

## Backgrounds

Parkinson's disease, Amyotropic Lateral Sclerosis and pose significant challenges in terms of treatment options [1]. These diseases are characterized by the progressive loss of neurons in specific regions of the brain, leading to debilitating symptoms and a decline in cognitive and motor functions [2]. Despite extensive research efforts, the development of effective therapeutics for neurodegenerative diseases has been impeded. One promising approach to address these challenges is the use of adeno-associated virus (AAV) gene therapy. AAV has emerged as a powerful tool for delivering therapeutic genes to target cells in a precise and controlled manner [3]. AAV vectors, especially AAV serotypes 2 and 9, can efficiently transduce neurons and be safe and well-tolerated in clinical trials for various neurodegenerative diseases [4, 5].

Recently, attempts have been made to treat a range of degenerative central nervous system (CNS) diseases by administering therapeutic genes into the brain and spinal cord through intracranial or intrathecal injections. Currently, several AAV-based gene therapies for Parkinson’s disease are in various stages of development and clinical trials [3, 6]. Prominent experiments utilizing neurturin (NRTN), glial cell line-derived neurotrophic factor (GDNF), and aromatic L-amino acid decarboxylase (AADC) are being investigated for PD gene therapies. These therapies aim to restore the function of dopaminergic neurons, alleviate PD symptoms, or impede disease progression. Gene therapy strategies also encompass the delivery of genes producing dopamine [7], genes modulating the basal ganglia circuitry to alleviate motor symptoms [8], and genes providing neuroprotection [9]. Notably, delivering survival genes, which encode proteins that play crucial roles in promoting cellular proliferation and protecting neurons from degeneration to the affected regions of the brain enhances neuronal survival and slows down the progression of neurodegenerative diseases. For instance, survival genes such as XBP-1 and AKT are recognized for their role in facilitating cell division [10, 11]. Analogously, they are identified as possessing the capacity to trigger tumorigenesis [12, 13]. Despite these potential adverse effects, clinical trials that directly administrates the survival genes targeting an array of neurodegenerative conditions, including Alzheimer’s, Parkinson’s, and Huntington’s disease, are actively being pursued [14–16]. While the results thus far are promising, they underscore a critical concern: the risk of tumorigenicity in unintended tissues related to the survival gene [17]. This poses an intricate challenge that demands resolution to minimize off-target effects instigated by the leakage of therapeutic transgenes into peripheral blood circulation [3, 17].

Precise control of transgene expression is crucial for ensuring the safety of AAV-based gene therapy for CNS neurodegenerative diseases, given AAV’s property to prolong the transgene expression for several years after injection [18]. In this study, we aim to overcome these limitations by developing a CNS specific AAV system that minimizes contamination of the transgene in the bloodstream. The strategy of introducing microRNA target sequence into the AAV vector to control off-target transgene expression in hematopoietic cells was utilized.

MicroRNAs (miRNAs) are initially transcribed as primary miRNAs (pri-miRNAs) and undergo processing in the nucleus by the drosha-DGCR8 complex into precursor miRNAs (pre-miRNAs), which are then processed in the cytoplasm by dicer into mature miRNAs [19]. These mature miRNAs are incorporated into the RNA-induced silencing complex (RISC) and guide it to target messenger RNAs (mRNAs) based on sequence complementarity, primarily within the mRNAs’ 3’ untranslated regions. This targeting either degrades the mRNA or inhibits its translation, effectively silencing gene expression [19]. Thus, the designing and encoding of the target sequence for a specific miRNA are anticipated to result in the downregulation of the corresponding mRNA expression.

In the present study, DX2, an antagonistic splicing variant of proapoptotic Aminoacyl-tRNA synthetase complex-interacting multifunctional protein-2 (AIMP2) was coded in the AAV vector system. Emerging research is beginning to illuminate the role of AIMP2 and its accumulation in the pathogenesis of PD [20]. AIMP2, a protein typically forming part of the multi-tRNA synthetase complex, plays a pivotal role in protein synthesis. Under specific conditions, AIMP2 can disassociate from this complex, leading to an aberrant accumulation of free form.

Recent study has revealed that excessive accumulation of AIMP2 contribute to neuronal cell death, a defining feature of PD. Notably, AIMP2 is a substrate of PARKIN. Mutant PARKIN is not able to degrade AIMP2. Thus, AIMP2 accumulation has been observed in the dopaminergic neurons of the substantia nigra in PD patients, implicating it in the neurodegenerative process associated with this disorder [21]. Also, AIMP2 physically binds to PARP-1 and leads to its aberrant activation. In dopaminergic neurons, increasing free AIMP2 seems to strongly induce PARP-1 induced neuronal death, so-called parthanatos [22]. But as AIMP2 is categorized an undruggable target due to its complex-forming and non-enzymatic character, direct targeting of AIMP2 seems to be difficult. DX2, a novel splicing variant of AIMP2, was initially identified by Choi et al. [23]. In contrast to the full-length AIMP2, which promotes cell death with its accumulation, DX2 appears to exert a survival protective effect on cells. Studies have shown that DX2 inhibits the cell death inducing activity of AIMP2, consequently promoting cell survival. The capability of DX2 to favor survival over death, particularly in the context of AIMP2 accumulation, offers profound implications for our understanding of PD and its potential treatment [24, 25].

## Methods

### Cell lines and reagents

Hela, Hek293T, SK-N-SH, SK-SH5Y, and THP-1 were obtained from the Korean Cell Line Bank (KCLB, Seoul, South Korea). Cells were grown in RPMI-1640, and supplemented with 10% fetal bovine serum (FBS) and 1% penicillin–streptomycin (HyClone, PA, USA). Rotenone was obtained from Sigma-Aldrich (MO, USA). 1-methyl-4-phenyl-1,2,3,6-tetrahydropyridine (MPTP) Hydrochloride was purchased from TCI chemicals (M2690, Tokyo, Japan) and dissolved in saline (0.9% NaCl, 3.75 mg/ml aliquots prepared fresh before injection). The chemicals used for neuronal cell death screening were: TNF-α (210-TA, Sigma-Aldrich, MO, USA), cycloheximide (66-81-9, MO, USA), actinomycin D (11805017, Thermo Fisher, MA, USA), cisplatin (D3371, TCI Chemical, Tokyo, Japan), paclitaxel (P1632, TCI Chemical, Tokyo, Japan), 5-fluorouracil (F0151, TCI Chemical, Tokyo, Japan), 6-hydroxydopamine hydrochloride (H4381, Sigma-Aldrich, MO, USA). The primary antibodies used were α-tubulin (sc-5286, Santa Cruz, TX, USA), Bax (2772 s, CST, MA, USA), tyrosine hydroxylase (ab6211, abcam, Cambridge, England), cleaved caspase-8 (9496 s, CST, MA, USA), cleaved caspase-9 (7237 s, CST, MA, USA), and p53 (sc-53394, Santa Cruz, TX, USA).

### Production and purification of scAAV2

To produce scAAV2-GFP and scAAV2-DX2, scAAV2-GFP and scAAV2-DX2 were generated by ligating oligonucleotides encoding the GFP or DX2 sequence into the pSF-AAV-ITR-CMV-ITR-KanR (Oxgene, Oxford, UK) vector. AAV viral vectors were prepared and transfected with AAV2 helper (OXGENE, Oxford, UK), AAV2 Rep-Cap (Oxgene, Oxford, UK), and pSF AAV-ITR-DX2 or pSF AAV-ITR-GFP in HEK293 cells. Media and cells were then collected and lysed.

To produce recombinant AAV serotype 2 (rAAV2) encoding DX2 or GFP at 40 L (20 L × 2) scales, 1 day before transfection of plasmid vectors, HEK 293 cells were counted (8 × 10^5^ cells/ml) and centrifuged at 300 × g for 7 min. The cell pellet was resuspended in HEK293 medium supplemented with 4 mM ultraglutamine and incubated for 24 h. Then, the DNA-PEIproHQ (transfection reagent) complexes (transfection mix) were generated according to the manufacturer’s recommendations. Briefly, two mixtures were generated in culture medium: (1) the plasmid DNA mix (scAAV2 DX2 or GFP—4 mg, rep/cap plasmid—7.2 mg, helper plasmid—8.8 mg, total 20 mg of DNA per 1 L of HEK293 medium with final 4 mM ultraglutamine) and (2) the PEIproHQ mix (60 mg of PEI per 1 L of HEK293 medium with final 4 mM ultraglutamine). These were combined and incubated for 10–15 min at room temperature. Finally, a volume equating to 10% of the production volume was added to each of the wave bags to be transfected (2 L of DNA-PEIproHQ complexes was added to 18 L of culture). Three days post-transfection, the cells were harvested, and lysed with lysis buffer containing 300 mM NaCl, 50 mM Tris–Cl, pH8.0, and 0.1% PF68. To remove the residual DNA, the lysate was incubated for 1 h at 37 ℃ after adding a nuclease. Then, we purified the AAV product using tangential flow filtration (TFF) and affinity chromatography, and the neutralized affinity chromatography eluate material was solubilized in a Tris-based buffer solution. In consideration of the effect of the AAV formulation on safety, the Tris-based buffer was changed to PBS-based buffer (PBS with 300 mM NaCl and 0.001% PF-68) by the 2nd TFF diafiltration to produce a high-titer virus vector stock. Part of the formulated (diafiltrated) viral particles was concentrated using 100 kDa Amicon Centricon^™^ and the concentrated products were sterilized by filtration using a 0.22 μm syringe filter (Sartorius). A final product (post-purification) viral genome (VG) titer of 4.05 × 10^13^ VG (1.62 × 10^13^ VG/mL, by qPCR) was obtained with this process.

### Phospho antibody array

SH-SY5Y cells were seeded at a density of 1 × 10^5^ cells per well. Subsequently, the cells were treated with 3 × 10^8^ vg/ml of AAV-GFP and AAV-DX2, respectively. After 72 h, the lysates were loaded on the membranes provided from Proteome Profiler^™^ Array Human Phospho-Kinase Array kit (ARY003C, R&D systems, MN, US) for detection. Quantification of each dot was performed using Image J software.

### Screening for organ-specific expression of AIMP2 and DX2

The hearts of 8-week-old male C57BL/7 mice were perfused using Phosphate-Buffered Saline (PBS). Subsequently, the individual organs were harvested. Following the lysis of each organ, the prepared protein supernatant was loaded onto a 10% acrylamide gel for Western blotting electrophoresis, before transferring the protein from the gel to a PVDF membrane; a 5% skimmed milk solution was then used for blocking. The membrane was incubated in primary antibody diluted with Tris-buffered saline (TBS) with 0.05% Tween (TBS-T) for 2 h. After three washes with TBS-T, the diluted secondary antibody was incubated for 1 h. Detection was performed after luminol (sc-2048, Santa Cruz, CA, USA).

### Screening miRNAs responsible to hematopoietic cells

Following the overexpression of myc-EV or myc-DX2 in THP-1 cells, we screened for microRNAs expressed in hematopoietic cell using the Mir-X miRNA First-Strand Synthesis Kit (Takara, Shiga-ken, Japan) as per the protocol specified by the kit. The miRNA forward sequences used for the synthesis were as follows: miR142-3p (gacagtgcagtcacccataa), miR22-3p (ggctgagccgcagtagttct), and miR122a-5p (ccttagcagagctgtggagt).

### Cloning of scAAV2-DX2 coding miR142-3p target sequences

Necessary components for plasmid amplification in Stbl3 competent cells, including the Kanamycin resistance gene and pUC and f1 ori elements, were obtained through Polymerase Chain Reaction (PCR) from pEGFP-N3. And the DX2 gene was amplified through PCR from pscAAV2-CMV2-DX2. The miR-142-3p target sequence was obtained through PCR using a DNA template inserted into a pUC57-Amp plasmid. Using the three PCR products, we performed restriction enzyme digestion and initiated an infusion reaction. The products were transformed into stbl3-competent cells. Colonies were then confirmed via colony PCR and sequencing, leading to the successful construction of the pscAAV-CMV-DX2-142-3p-TS vector. The primers used were as follows: Forward (Insert_AccI): ggcccgcatgcgtcgac cttaaggcgtaaattgtaagc, Reverse (Insert_BgIII): gaagctttctagatct ttttccataggctccgcc, Forward (DX2_EcoRI): ctttggaactgaattc atgccgatgtaccaggta, Reverse: GAGCTAGCAAGCTTCTGG cttaaggagcttgag, Forward: ctcaagctccttaag CCAGAAGCTTGCTAGCTC, Reverse (3p-TS_BamHI): gatatctcggatcc CTACAAGCTTTGTAGTG.

### Luciferase assay using the miRNA target sequence

HEK293T cells were seeded in a 96-well plate at a density of 8 × 10^4^ cells/well. After overnight culture, the cells were transiently transfected with plasmids using Lipofectamine 2000 (Invitrogen, CA, USA). In each transfection, 200 ng of vector and 25 μM of each miRNA (miR122a-5p, miR142-3p and miR22-3p) and *Renilla* luciferase plasmid were used as indicated. Cells were incubated in the presence of the indicated reagents for 48 h, and luciferase activity was measured using a Dual-Glo luciferase assay kit (Promega, WI, USA) according to the manufacturer’s instructions. Each test condition described was represented by three replicate plates, and statistical significance values are calculated after normalizing to *Renilla* luciferase activity to determine transfection efficiency.

### Luciferase assay using the miRNA target sequence

THP-1 cells were seeded in a 6-well plate at a density of 5 × 10^5^ cells/well. The cells were infected with AAV-DX2-miR142-TS at 2000 MOI. After 24 h, The cells were transfected with pre-designed miR142 specific inhibitor (SMI-001, Bioneer, seoul, South Korea) using Lipofectamine^™^ RNAiMAX Transfection Reagent (Thermo Fisher Science, CA, USA). After 48 h, Cells were collected and RNA isolation was performed.

### Surgical procedures

All mice experiments were performed under the Kyung Hee University Institutional Animal Care & Use Committee guidelines (KHUASP(SE)-18-101). To infuse scAAV2-GFP or scAAV2-DX2, male c57bl/6n mice aged 8 weeks were injected with scAAV2-GFP or scAAV2-DX2 in the right striatum. Each mouse was ethically anesthetized using ketamine and a muscle relaxant. The anesthetized mice were then placed on the stereotaxic device with tooth and ear bars. The total volume of virus per mouse was 3 μl. The injection was performed using a Hamilton syringe at the following coordinates: AP: + 0.5 mm, ML: + 1.8 mm, DV: − 3.7 mm. In the MPTP group, male c57bl/6n mice aged 8 weeks received 30 mg/kg of MPTP subcutaneously to recreate a reference model as described by Petroske et al. [26]. All animals received a total of 10 injections consecutively for 5 days. Following the treatment, all animals were fed sucrose solutions. To evaluate the efficacy of DX2 in the MPTP mice model, scAAV2-DX2 and scAAV2-GFP were injected into the substantia nigra. The injection was performed using a Hamilton syringe at the following coordinates: AP: − 2.7 mm, ML: ± 1.5 mm, DV: − 4.5 mm. All stereotaxic injections were performed at a rate of 0.5 μl/min and the needle was removed out of place for another 10 min after the injection before slowly being removed.

Utilizing 8-week-old male C57BL/6 mice, an intrathecal injection was performed into the lumbar region of the spinal cord with an scAAV2-DX2 virus at a concentration of 1.6 × 10^10vg/ul and a total volume of 3 ul, using a 30 G Hamilton syringe. The accuracy of the injection was confirmed through the observation of tail movement in the mice during the procedure. Three weeks post-injection, blood was isolated from the posterior vena cava region using 25G polypropylene syringes, and the lumbar region of the spinal cord was isolated through dissection.

### DNA isolation and analysis from mouse samples

To generate a standard curve, pSE-scAAV2-DX2 plasmid (2.035 × 10^11^ copies/μl) was used as a standard. The plasmid was diluted with nuclease-free water to a concentration of 2 × 10^9^ copies/μl and subjected to serial dilutions for the standard curve. For DNA isolation, organs stabilized in RNAprotect Tissue Reagent (Cat Nos. 76104 and 76106, QIAGEN, Hilden, Germany) were thawed at room temperature. Buffer RLT Plus (β-ME + DX) was added to the organs. DNA isolation was performed using the AllPrep DNA/RNA/miRNA Universal Kit (Cat No. 80224, QIAGEN, Hilden, Germany), following the kit’s recommended protocol.

### RNA isolation and analysis from mouse samples

To generate a standard curve, ivtRNA (1.471 × 10^12^ copies/μl) was used as a standard single-stranded RNA (ssRNA). The ssRNA standard was diluted with nuclease-free water to a concentration of 1011 copies/μl. For RNA isolation, the same AllPrep DNA/RNA/miRNA Universal Kit (Cat No. 80224, QIAGEN, Hilden, Germany) was used, following the recommended protocol for isolating RNA from the organs. For the detection of scAAV2-DX2, quantitative PCR (qPCR) was performed using the following primers; Forward: 5′-CTGGCCACGTGCAGGATTA-3′, Reverse: 5′-GACAGGACCCTGAAGTGCTC-3′.

### The SHIRPA test

For a better understanding of subtle phenotypical changes in mice, the SHIRPA (SmithKline Beecham, Harwell, Imperial College, Royal London Hospital; phenotype assessment) was performed. The SHIRPA test is a semi-quantitative analysis based on the primary method developed by Rogers et al. [27]. Neuro-behaviour assessment including tremor, grooming, spontaneous activity, locomotor activity, and clasping was measured during each set of tests. Physical phenotype assessment including palpebral closure, piloerection, body position, and tail position was also measured. Every mouse in the MPTP model group was behaviourally tested. All analyses were performed by an experimenter blinded to the group. Behaviour tests were conducted at different points after the AAV injection.

### Rotarod test

To examine the motility of the MPTP and rotenone mouse models, a rotarod test was conducted at 3 and 15 days post-lesion. All mice were trained on the same machine with equal rotating speed to show a stable performance. Training procedures consisted of three sessions on 2 days in which each session included three individual trials, lasting at least 200 s each. All mice were trained at 5, 10, and 15 rpm (revolutions per minute). The final test was conducted at 15 rpm (three sessions, 300 s each) on the third day. To reduce fatigue, each mouse was given at least 5 min of rest between each measurement.

### Tissue preparation

At the end of the behavioural assessment, 7 weeks for the MPTP model post-lesion. Mice were sacrificed after being anesthetized with isoflurane and perfused with 0.9% saline mixed with 4% paraformaldehyde (PFA; Yakuri Pure Chemicals) in phosphate buffer (PBS) at a pH of 7.4. Organs including the lungs, heart, liver, kidney, ovary, spleen, bladder, spinal cord, and brain were collected. The brains were fixed overnight in 4% PFA followed by a dehydration step with 30% sucrose in PBS. Brain samples were then frozen with optimum cutting temperature compound (O.C.T. compound; 4583, Sakura Finetek, CA, USA) and cut on a cryotome (Leica CM1850; Leica Biosystems, Wetzlar, Germany) into 30 μm thick coronal sections through the entire substantia nigra and striatum. All sections were made from the posterior end of the SN to the anterior end of the striatum. The sections were stored at – 20 ℃ in a cryoprotectant solution made with ethylene glycol, glycerol, and 0.2 M phosphate buffer.

### Immunofluorescence staining

For the staining, the floating brain tissues in cryoprotectant solution or attached cells on a coverslip were moved to a slide glass. After washing with PBS three times for 5 min, blocking with 5% BSA in PBS was performed for 30 min followed by permeabilization with 0.2% triton × 100 in PBS. The primary antibody was diluted with 1% BSA in PBS, applied to the section/cells, and incubated at room temperature for 1 h. After three washes with PBS-T (0.1% tween in PBS) for 5 min, the secondary antibody diluted with 1% BSA in PBS was incubated in a dark room for 1 h. Mounting with VECTASHILD antifade mounting medium with DAPI was used for nucleus counterstaining.

### Image analysis

Images were analyzed with Image J (http://rsb.info.nih.gov/ij) using the Time Series Analyzer plugin, accessible at https://imagej.nih.gov/ij/plugins/time-series.html. Synaptic boutons of neurons were appointed as oval regions of interest (diameter, 10 pixels), and the amplitude of pHluorin-based fluorescence at synapses was counted using Origin Pro 2020. The kinetics of endocytosis was measured using a single exponential decay function.

### Statistics analyses

The *p* values are represented for mean ± SEM and data were statistically analyzed using Student’s *t*-test or ANOVA, where appropriate. A *p*-value of less than 0.05 was considered to be statistically significant.

## Results

### Enhancement of neuronal survival signal by AAV-DX2

**Fig. 1:**
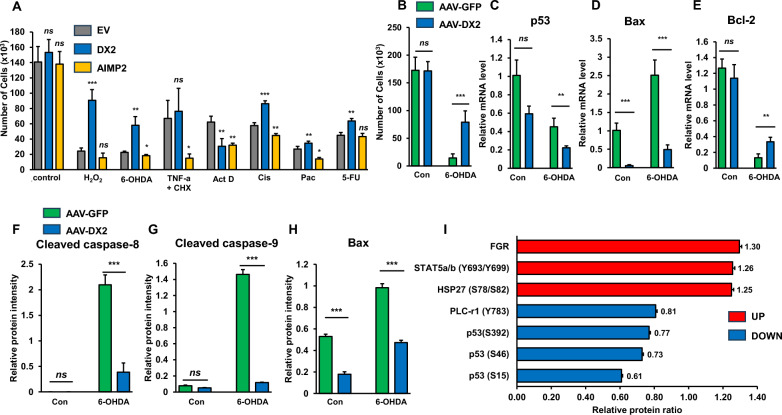
Protective effect of DX2 against ROS-induced cell death in SH-SY5Y cells. **A** A graph showing a viable cell counting in SH-SY5Y cells overexpressing Flag-EV, Flag-AIMP2, and Flag-DX2, after screening with cell death-inducing chemicals. All chemicals were treated for 24 h after vector overexpression, at the following concentrations: H_2_O_2_ (100 μM), 6-OHDA (100 μM), TNF-α (10 μM), cycloheximide (CHX, 10 μM), actinomycin D (Act D, 10 nM), cisplatin (Cis, 10 μM), paclitaxel (Pac, 10 μM), and 5-fluorouracil (5-FU, 10 μM). **B** A graph comparing cell counts between AAV-GFP and AAV-DX2 infections. All cell countings were repeated three times. **C**–**E** Quantitative PCR graphs analyzing the expression patterns of mRNAs involved in cell survival by AAV-DX2 **F**–**I** Immunoblot assays confirming the Caspase-cascade pathway in cell survival induced by AAV-DX2. **J** Quantification of dot blot assay data for AAV-GFP and AAV-DX2

To elucidate which cell death pathway is most affected by the anti-apoptotic function of DX2, we treated various apoptotic stress on the neuroblastoma cell line SH-SY5Y. As described in previous papers [24, 25], while AIMP2 induced cell death commonly under treated condition with most cell death inducing agents, DX2 effectively suppressed apoptosis (Fig. 1A). Of note, overexpression of DX2 most effectively mitigated cell death induced by oxidative stress such as H_2_O_2_ and 6-OHDA treatments (Fig. 1A). Next, we introduced an AAV-DX2 into the cells, to achieve a more stable overexpression of DX2. Robust expression of DX2 with the introduction of AAV-DX2 was confirmed (Additional file 1: Fig. S1). To determine how effectively AAV-DX2 inhibits neuronal cell death, we conducted cell death assays under 6-OHDA treated condition. AAV-DX2-infected cells exhibited a significantly higher cell survival compared to those incubated with the control virus, AAV-GFP (Fig. 1B). Under the same experimental conditions, we examined the mRNA levels of cell death markers. We observed a significant decrease in p53 (Fig. 1C) and Bax (Fig. 1D) across AAV-DX2 treated samples, along with a reversal of Bcl-2 reduction typically induced by cell death signals (Fig. 1E). The 6-OHDA-induced cleaved form of caspase-8 (Fig. 1F and Additional file 1: Fig S2) and caspase-9 (Fig. 1G and Additional file 1: Fig S2) were also reduced by AAV-DX2. Protein level of Bax was also negatively affected (Fig. 1H and Additional file 1: Fig S2).

To further explore the neuronal survival signal pathways mediated by AAV-DX2, we infected AAV-GFP or AAV-DX2 into SH-SY5Y cells, followed by a phospho-proteomic analysis (Additional file 1: Fig. S3). In screening the phosphorylation of 39 proteins, we found that most of the proteins showing a prominently increased phosphrylation in AAV-DX2-treated cells compared to AAV-GFP, were cell survival-associated genes such as FGR, STAT5 and HSP27 (Fig. 1I. See red bar graphs). Contrastly, cell death marker p53 phosphorylation was significantly reduced in AAV-DX2 treated cells (Fig. 1I. See blue bar graphs).

### Optimization and verification of AAV2-mediated delivery system

**Fig. 2:**
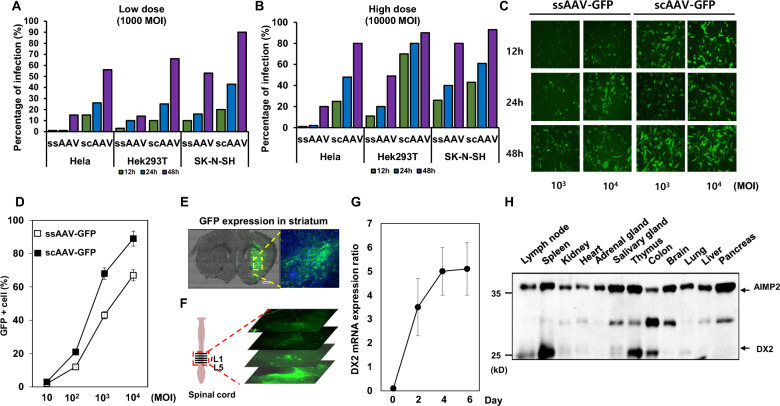
Efficacy test of scAAV2-GFP and scAAV2-DX2 in vitro and in vivo **A**, **B** Transduction efficacy test of AAV2-GFP in Hela, Hek293 and SK-N-SH. **A** 1000MOI concentration of ssAAV2-GFP and scAAV2-GFP **B** 10000MOI cencentration of ssAAV2-GFP and scAAV2-GFP **C** GFP expression was observed with fluorescence microscopy in SH-SY5Y. **D** The percentage of GFP-positive cells was measured following dose-dependent infection for 48 h with ssAAV2-GFP and scAAV-GFP in the SH-SY5Y cell line. **E** scAAV2-GFP expression in the right striatum, scale bar: 500 μm and 100 μm**. F** scAAV2-GFP expression in the spinal cord of intrathecally injected mice. The lumbar region specified as L1 to L5 was injected and used for confocal imaging. **G** DX2 expression was checked on the day after injection at the isolated substantia nigra using real-time qPCR. After scAAV2-DX2 treatment in H_2_O_2_-induced apoptosis situations. **H** The difference in AIMP2 and DX2 expression levels in mouse organ

The single-strand adeno-associated virus, or ssAAV2, carries a single-stranded DNA. This necessitates conversion into a double-strand by the host cell's machinery before the gene can be expressed, potentially causing a delay in gene expression. On the other hand, the self-complementary adeno-associated virus, scAAV2, contains a double-stranded DNA. This bypasses the need for conversion in the host cell [28]. To determine which viral system to use for efficient infection between ssAAV2 and scAAV2, green fluorescent protein (GFP) sequences was inserted to both viral system and we administrated to three cell lines including human neuroblastoma cell line, SK-N-SH, infected with 1000 and 10000 multiplicity of injection (MOI). As shown in Fig. 2A, B, In Hela and HEK293 cell lines, the infection ratio after infecting with ssAAV at 1000 MOI for 48 h was similar to the infection rate observed when treated with scAAV at the same MOI for 12 h. In the case of SK-N-SH cell lines, the infection ratio after treating with ssAAV at 1000 MOI for 24 h was comparable to the infection rate when treated with scAAV at 1000 MOI for 12 h (Fig. 2A). This phenomenon also showed a similar trend when treated at 10000 MOI (Fig. 2B). This allowed us to conclude that scAAV2 was more effective at infection than ssAAV2 in all tested cell lines.

The SH-SY5Y cell line, which is widely used in Parkinson's disease (PD) research, is known to best reflect the characteristics of dopaminergic neuronal cells [29], and, we also confirmed that scAAV2 also infected SH-SY5Y more efficiently than ssAAV2 (Fig. 2C, D). Significant difference in the level of injection was observed depending on the cell type as shown in Fig. 2A, B.

In order to evaluate the scAAV2 viral system infectivity to the striatum region—a primary target site in Parkinson’s disease—we administered scAAV2-GFP via intracranial injection, targeting the right portion of the striatum region, through a minimally invasive procedure. Initial verification of the transgene expression was achieved through the detection of the GFP signal in striatum region (Fig. 2E). Next, infectivity of the scAAV2 viral system to the spinal cord, another main structure of the CNS, was also evaluated. We targeted the lumbar region of a mouse model, employing an intrathecal injection of the scAAV2-GFP virus. Intense GFP signal around the spinal cord injection site suggested an observable spreading of GFP signaling from the focal point of administration (Fig. 2F). To confirm whether DX2 serves as an effective therapeutic target for Parkinson's disease (PD), we generated scAAV2-DX2 (self-complementary Adeno-Associated Virus-DX2). For further validation of DX2 expression at the brain injection site, we delivered the scAAV2-DX2 virus to the substantia nigra, a region densely populated with dopaminergic neurons, via an intracranial injection. The expression analysis was conducted every two days following the injection. Significantly, DX2 expression reached a saturation point four days after the in vivo administration of the viral vector in the intracranial region (Fig. 2G). The expression level of AIMP2 and DX2 in the tissues was determined with western blotting using a monoclonal antibody to detect both types of AIMP2 and DX2. While AIMP2 seemed to be expressed broadly and stably throughout the tissues, DX2 expression showed fluctuation between tissues (Fig. 2H). Especially, immune or hematopoietic cell-rich organs such as the thymus, spleen displayed relatively high expression of DX2 compared with other tissues (Fig. 2H, lower bands). DX2 endogenous expression was rarely observed in the brain. In light of these phenomena, we aimed to construct a system to regulate the rampant expression of survival genes in organs rich in hematopoietic cells.

### Strategic implementation and evaluation of miR142-3p-mediated suppression of DX2 in hematopoietic cells

**Fig. 3:**
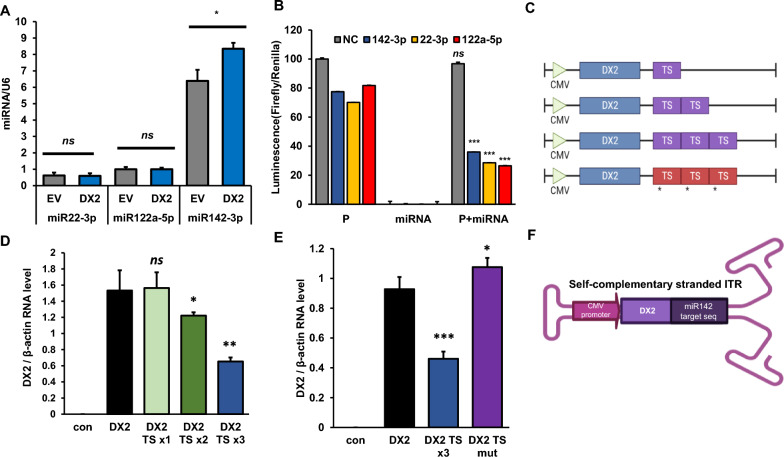
Development of scAAV-DX2 with the microRNA142-3p target sequence **A** Screening of hematopoietic cell expressed microRNA. Among the miRNA, miR142-3p was more highly expressed than the others **B** The relative value of Luciferase assay result with hematopoietic cell expressed miRNA and its target plasmid. *P* Plasmid. **C** Scheme of DX2 vector with variously repeated miR142-3p target sequence and mutation form. The symbol of * is a single mutation sequence. **D, E** DX2 expression is inhibited by the miR142-3p target sequence in hematopoietic cells. **F** Scheme of scAAV-DX2 with miR142-3p target sequence. *ns*: no-significant; *: *p* < 0.05; **:*p* < 0.01; ***:*p* < 0.001. *t*-test. TS: miR142-3p target sequence; mut: mutation

In an attempt to suppress unexpected transgene expression in hematopoietic cells, we integrated the target sequence (TS) of a microRNA (miRNA or miR) into the vector system. miRNAs that are specifically expressed in hematopoietic cells was conducted. miRNA, which is not expressed in CNS but is specifically expressed in hematopoietic cells, meets our vector system criteria for expressing the therapeutic gene exclusively in the CNS. Accordingly, we conducted a search based on this criterion and identified the following miRNA candidates for selection: miR22-3p, miR122a-5p, and miR142-3p. Then, we evaluated the expression level of each miRNA candidate, miR22-3p, miR122a-5p, and miR142-3p, following DX2 overexpression. Among the three candidate miRNAs, miR142-3p was noted to be abundantly expressed in THP-1 leukemia cells and its expression notably escalated upon DX2 overexpression (Fig. 3A). Next, we screened the optimal target sequences for each hematopoietic cell-specific miRNA (data not shown). We observed that all miRNA TSs displayed a decrease in luminescent output in the presence of their corresponding miRNAs (Fig. 3B). Collating these findings, we resolved to incorporate the miR142-3p-TS in our experimental design. To assess the effect of miR-142-3p-TS in the vector on DX2 expression and to find out the optimal repetition number of miR-142-3p-TS in the vector for inhibition of DX2 expression, three constructs were made containing 1, 2 and 3 miR-142-3p-TS.

Schematic of miR142-3p-TS constructs are shown in Fig. 3C. Inhibition of DX2 expression in vector transfected HEK293 cells was observed with the miR142-3p × 1 repeat (100 pmol) miR142-3p-TS and as the number of core binding sequence in miR142-3p-TS are increased, miR142-3p inhibition on DX2 expression was also increased (Fig. 3D). The TS × 3 core sequence containing vector showed significant inhibition, whereas no inhibition was observed for the mutated 3 × sequence, vector with a single point mutation (the sequence: 5′-TCCATAACGTAGGAAACACTACA-3′) (Fig. 3E). Given with these findings, the miRNA142-3p TS was inserted preceding the 3’ ITR in scAAV2-DX2 vector system (Fig. 3F).

### Selective targeting and miR142-3p-dependent modulation of scAAV2-DX2 expression

**Fig. 4:**
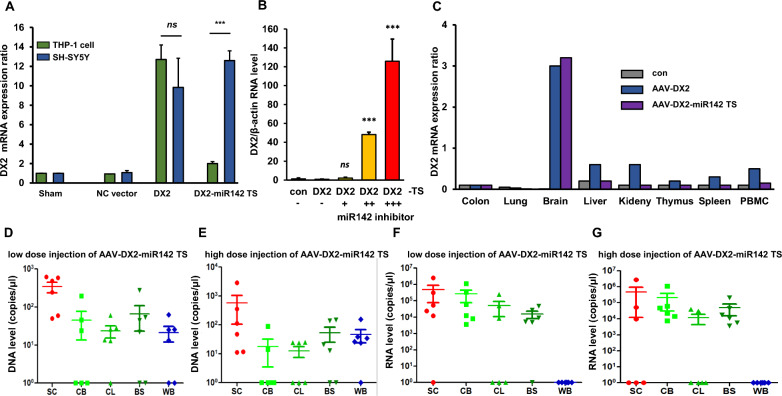
scAAV2-DX2-miR142-3p target sequence selectively expressed in the target organ **A** Comparison of transgene expression between scAAV-DX2 and scAAV2-DX2-miR142-3p target sequence plasmid in hematopoietic cells and neuronal cells. **B** Relative expression ratio of DX2 with or without miR142 inhibitor in THP-1 cells. ‘ + ’ means 10 pmol of miR142 inhibitor. **C** Body distribution test for scAAV-DX2 in intracranially injected mice. **D**–**G** Assessing scAAV-DX2 spreading and expression during intrathecal injection administration between lumbar and blood. **D** DNA level of DX2 in low-dose injected mice **E** DX2 level of DX2 in high-dose injected mice. **F** RNA level of DX2 in low-dose injected mice **G** RNA level of DX2 in high-dose injected mice. animal number: n = 6, Low dose: 2.4 × 10^10^vg/animal, High dose: 9.6 × 10^10^vg/animal. *ns*: no-significant; *: *p* < 0.05; **:*p* < 0.01; ***: *p* < 0.001 *TS* miR142-3p target sequence, *SC* spinal cord, *CB* celebellum, *CL* cerebral lobes, *BS* Brain stem, *WB* whole blood

To check the neuron specific expression of DX2 of the miR142-3p-TS in our AAV vector system, THP-1 monocytic cells and a neuroblastoma SH-SY5Y cell line were treated with void vector, scAAV2-DX2 or scAAV2-DX2-miR142-3p-TS. While DX2 was expressed similarly between THP-1 and SH-SY5Y cells, the expression of DX2 was significantly suppressed only in THP-1 cells with miR142-3p-TS (Fig. 4A). Additionally, to ascertain whether the suppression of AAV-DX2-miR142-3p TS expression in the THP-1 cell line is indeed due to the function of miR142, we examined the expression of the viral gene in the context of miR142 knockdown. The results revealed that as the concentration of the miR142 inhibitor was increased, there was a corresponding increase in DX2 expression in THP-1 cells (Fig. 4B).To assess the effect of miR-142-3p-TS in the vector on DX2 biodistribution of our vector system, three groups of female mice were administered vector [NC vector, scAAV2-DX2 without miR142-3p-TS and scAAV2-DX2-miR142-3p-TS] via stereotaxic intracranial injection into the substantia nigra. qPCR analysis confirmed that the expression of DX2 specifically increased only in the brain tissue in the scAAV2-DX2 and scAAV2-DX2-miR142-3p-TS groups (Fig. 4C). In the scAAV2-DX2 group, DX2 mRNA levels were detectable in peripheral blood mononuclear cells (PBMCs) and several PBMC-retaining tissues, such as the spleen and kidney. This expressions in untargeted tissues were completely suppressed to the baseline level in the scAAV2-DX2-miR142-3p-TS groups (Fig. 4C).

Prior reports have suggested that, when utilizing AAV gene therapy with local injections, the intended gene does not express exclusively at the site of injection but distributes throughout the entire organ [30]. To further examine the biodistribution pattern of our miR142-3p-TS inserted scAAV2-DX2 construct, we performed intrathecal injections into the lumbar region of the mouse spinal cord. First and foremost, to examine the toxicity of scAAV2-DX2-miR142-3p-TS, we measured various indicators in the mouse body weight and serum (AST, ALT, ALP, LD, CK, GLU, BIL UN, CRE, CHO, TG, PL, IP, CA, NA, K, CL, TP, ALB and A/G, Additional file 1: Table S1) and separately collected urine for urinalysis measurements. As a result, no toxicity was found in dose-dependent body weight (Additional file 1: Table S2), serum analysis (Additional file 1: Table S3), and urinalysis (Additional file 1: Table S4) of scAAV2-DX2. Furthermore, upon conducting H&E staining for histopathology analysis of the injection site, the dorsal root ganglion (DRG) region, no abnormalities were found compared to the control (Additional file 1: Fig. S4). Subsequently, we isolated the injected lumbar region and various brain sections (Cerebellum, Cerebral lobes, brain stem), as well as blood, and quantified the levels of DX2 DNA and RNA. Our findings revealed that while DNA was detectable in both the brain, including the injection site, and blood (Fig. 4D, E), RNA was only identified in the injection site and various sections of the brain, with no detection in the blood (Fig. 4F, G). These results suggest that the scAAV2-DX2-miR142-3p-TS viral system is degraded at the RNA level, not at the DNA level, indicating an effect attributed to the miR142-3p-TS.

### The preventive and therapeutic effect of scAAV-DX2 in the MPTP-induced PD model

**Fig. 5:**
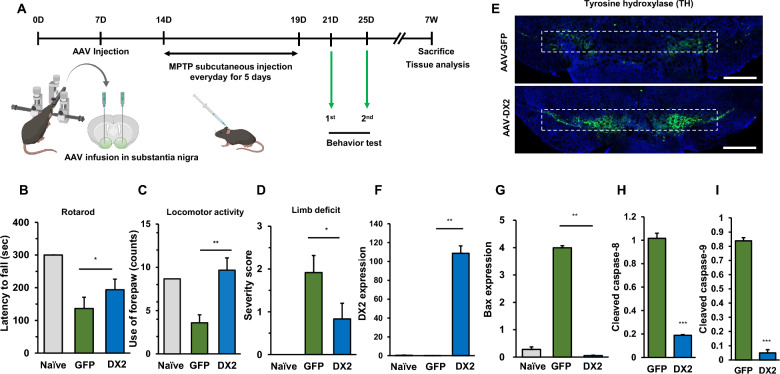
DX2 restores motor symptoms in MPTP-induced PD model **A** Scheme of scAAV2-DX2 transduction in MPTP-induced mouse model. scAAV2-DX2 was infused in the substantial nigra region with the stereotaxic machine and then subcutaneous injection with MPTP was performed once per day. Before measuring the behaviour test, the training of each test was continued. **B** scAAV2-DX2-treated mice showed slightly longer latency to fall in the rotarod test when compared with vehicle (scAAV2-GFP, GFP), indicating that scAAV2-DX2 attenuated damage towards dopaminergic neurons. **C** DX2-treated mice showed improved locomotor activity based on the SHIRPA test. **D** DX2-treated mice showed a relatively lower level of limb deficit. **E** Immunofluorescence image of TH-positive cells in the mouse substantia nigra. The white scale bar represents 500 µm **F**, **G** DX2 **F** and Bax **G** mRNA expression of the indicated mice brain. **H**–**I** Quantification of immunoblot assay for cleaved caspase-8 and cleaved caspase-9. animals: naive, n = 6; GFP, n = 9; DX2, n = 12. scAAV2: scAAV2-GFP, 4 × 10^9^ vg; scAAV2-DX2, 4 × 10^9^ vg. *ns*, non-significant; *, *P* < 0.05; **, *P* < 0.01; ***, *P* < 0.001; *t*-test

Prior research has demonstrated that the neurotoxin MPTP induces significant dopaminergic neuron death, prompting us to investigate the impact of DX2 on an MPTP-induced Parkinson’s disease (PD) model (Fig. 5A). Notably, scAAV2-DX2 injected mice exhibited increased fall latency and enhanced locomotor activity in comparison to naïve mice, as evidenced by the open field test (Fig. 5B, C). Furthermore, the elevated body swing (EBS) test revealed a significant reduction in limb deficit scores in scAAV2-DX2 treated mice compared to scAAV2-GFP treated mice (Fig. 5D). Collectively, these findings indicate substantial improvements in motor behavior in scAAV2-DX2 injected mice. Tyrosine hydroxylase (TH) is a crucial marker for dopaminergic neurons. In mice injected with AAV-DX2, a significant recovery of TH-positive cells in the substantia nigra region was observed compared to control mice (Fig. 5E). Moreover, elevated DX2 levels were detected in the substantia nigra region (Fig. 5F), accompanied by a decrease in the apoptotic marker Bax (Fig. 5G) in DX2-transduced mice brains. Additionally, measuring the protein levels of caspase-8 (Fig. 5H and Additional file 1: Fig S5) and caspase-9 (Fig. 5I and Additional file 1: Fig S5) in the substantia nigra region, we observed a decrease in both cleaved forms in areas where AAV-DX2 was administered.

### In vivo* efficacy of scAAV2-DX2 in rotenone-induced Parkinsonism*

**Fig. 6:**
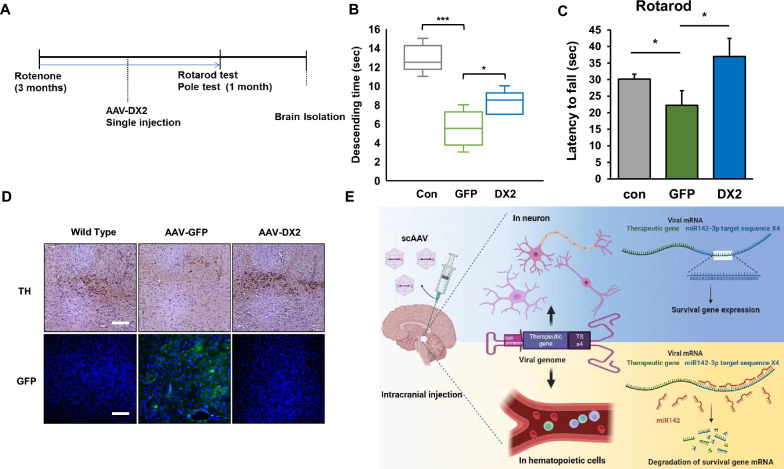
scAAV2-DX2 has a therapeutic effect in rotenone-induced parkinsonism mice** A** scheme of the therapeutic model of rotenone-induced Parkinson’s disease using scAAV2-DX2 **B** The pole test. **C** The rotarod test. scAAV2-DX2 recovered motor coordination and balance in the rotenone-treated PD mouse model. **D** Immunohistochemistry and immunofluorescence image of the mouse substantia nigra. The upper panel shows TH-positive cells in the striatum and the lower panel indicates the distribution of an injected-GFP expressing virus. The white scale bar represents 50 µm. Animals; n = 5 (in each group), ns, no significant; *, *P* < 0.05; ***, *P* < 0.001; *t*-test. **E** Schemic image of regulation of therapeutic viral genome

To investigate the in vivo efficacy of this approach in preventing cell death, we proceeded to directly inject scAAV2-DX2 into the substantia nigra of rotenone-induced Parkinsonism animals (Fig. 6A). To identify potential therapeutic effects in our mouse model, we administered the vertical pole test, observing an amelioration in the behavior of scAAV2-DX2-transduced mice, which was associated with a reversal of the neuronal damage caused by rotenone (Fig. 6B). In a similar vein, results from the rotarod test demonstrated superior motor capabilities in mice treated with scAAV2-DX2 (Fig. 6C). To directly observe the recovery of dopaminergic neurons influenced by DX2, we stained the substantia nigra region in mice with Tyrosine hydroxylase (TH), a marker for dopaminergic neurons. We were able to confirm that damaged dopaminergic cells in the brains of rotenone-treated mice exhibited significant recovery in scAAV2-DX2-injected mice (Fig. 6D).

Taken together, a miR142-3p-TS insertion into AAV vector system at 3’ UTR of viral genome completely blocked the DX2 being expressed in hematopoietic cells, the major population of of non-neuronal cells in the injected tissue area. Suppressing the expression of the DX2 in hematopoietic cells and targeting only to neuronal cells in injected tissues can be achieved by insertion of miR142-3p into the AAV vector, as miR142-3p is expressed only in hematopoietic cells (Fig. 6E).

## Discussion

Our study demonstrates a CNS-specific gene delivery methodology employing miRNA target sequence-coded adeno-associated virus (AAV) system as a novel therapeutic avenue for degenerative Central Nervous System (CNS) diseases. We have particularly illustrated the therapeutic efficacy of the survival gene DX2, dispatched via this miRNA target sequence-coded AAV2 delivery vector in Parkinson’s Disease (PD) animal models (Figs. 5, 6).

Our study includes a side-by-side comparative analysis of the tissue distribution of introduced transgenes in AAV injected mouse with miRNA142-TS encoded AAV2*,* the serotype with neurotropism [31], injected mouse via intracranial administration. This comparative approach provides valuable information on the systemic distribution potential and its distribution patterns, even when AAV is administered via local injection (Fig. 4B).

The engineered AAV system, as portrayed in our study, offers a level of precision that ensures the introduced transgene is expressed solely at the site of injection, precluding transgene expression in blood cells or unintended tissues. This illuminates significant information on the tissue distribution of AAV, when it is delivered to the CNS via intrathecal and intracranial injections.

One of the key challenges of the present study was the precise regulation of therapeutic transgene expression in targeted neuronal tissue. This concern arises from the dual nature of survival genes, which can promote cellular proliferation and protect neurons from degeneration, but may also pose a risk of tumorigenicity in unintended tissues.

Our experimental procedures have consistently ascertained that DX2 itself is non-tumorigenic and exhibits neither adverse nor immunogenic systemic effects (Additional file 1: Fig. S2 and Table S3). Nonetheless, achieving precise regulation of transgene expression in the intended target tissue, irrespective of whether the introduced transgene is a survival gene, is crucial for ensuring long-term clinical application safety.

Addressing these concerns, we incorporated a microRNA target sequence into our scAAV-DX2 construct. We harnessed miR142-3p, predominantly expressed in hematopoietic cells [32–34], to inhibit off-target DX2 expression in these cells. The successful realization of this miRNA-mediated suppression strategy underscores the potential of miRNAs in bolstering the specificity and safety of AAV-based gene therapy. Our miR142-3p target sequence, 5′-TCCATAAAGTAGGAAACACTACA-3′, effectively quelled RNA expression of the target gene in tissues where viral DNA was detected, indicating that miR142-3p provokes RNA breakdown [35–37]. Notably, the inclusion of a triple repeat of the target sequence magnified the efficacy (Fig. 3D, E). Among a range of hematopoietic cell expressed miRNAs evaluated, including miR22-3p [38], miR122-5p [39] and miR142-3p was deemed sufficiently robust to suppress the target gene, with DX2 overexpression leaving its expression level unaffected.

Our results showcase that the self-complementary AAV (scAAV) delivery system outperforms its single-stranded counterpart in delivering DX2 to neurons compromised by PD. This finding amplifies our understanding of the advantages of scAAV vectors in delivering therapeutic transgenes, particularly in the milieu of neurodegenerative diseases where efficient gene delivery to neuronal cells is paramount. Moreover, the scAAV2-DX2 delivery system proved effective both in vitro and in vivo, successfully infiltrating the striatum region and substantia nigra in a mouse model (Figs. 5D, 6F). These regions, characterized by the loss of dopaminergic neurons, are of significant interest in PD. The capacity of our scAAV2-DX2 construct to effectively express in these critical regions underscores its potential in augmenting neuronal survival and mitigating PD symptoms.

In terms of therapeutic efficacy, scAAV2-DX2 illustrated significant improvements in motor behavior in two distinct PD mouse models: the rotenone-induced (Fig. 5B, C) and MPTP-induced PD models (Fig. 6B–D). Immunostaining results buttressing these findings showed the restoration of damaged dopaminergic cells in the brains of mice treated with scAAV2-DX2. The capability of our scAAV2-DX2 construct to elicit therapeutic effects in these diverse PD models bolsters its potential as a treatment modality for PD. The promising outcomes from this study posit that, with further optimization and safety evaluations, AAV-mediated delivery of DX2 could prove a viable therapeutic approach for PD and potentially other neurodegenerative diseases.

In summation, our research underscores the potential of AAV-based gene therapy, with a specific focus on the scAAV2-DX2 construct, for targeted delivery of survival genes to neurodegenerative regions in a controlled and efficient manner. The addition of miRNA-mediated suppression bolsters specificity. The antagonistic interplay between AIMP2 and DX2 illuminates new possibilities for therapeutic strategies targeting PD. Continual studies and development along these lines may herald advancements in PD treatment.

## Conclusions

This study has successfully demonstrated the development and optimization of an AAV-based gene delivery system for CNS disease gene therapy. We confirmed its target specific transgene delivery efficiency in neuronal cells and NS tissues, with minimal off-target effects in hematopoietic cells, which was achieved through the integration of miR142-3p target sequence. The vector system proved effective in delivering the therapeutic gene DX2, resulting in significant neuroprotective effects. It improved motor behavior and reduced neuronal death markers in animal models of Parkinson's disease. In summary, our study suggests the therapeutic protential of this innovative scAAV2-DX2-miR142-3p target sequences vector system for treating neurodegenerative diseases including Parkinson’s disease.

### Supplementary Information

**Additional file 1: Fig S1.** Expression of AAV-DX2 in SH-SY5Y. SH-SY5Y cells were infected with AAV-GFP and AAV-DX2. Total RNA in transfected cells were analyzed by quantitative RT-PCR. **Fig S2.** Immunoblot assay of cell survival efficacy by AAV-DX2. SH-SY5Y cells were infected with AAV-DX2. SH-SY5Y cells were infected with AAV-GFP and AAV-DX2. Cleaved-caspase-8 and Cleaved-caspase-9 were detected. **Fig S3.** Comparison of cellular signaling changes of AAV-DX2 using Dot Blot assay. **Table S1.** Abbreviation of parameters of clinical chemistry. **Table S2.** Comparison of body weight changes between the AAV-DX2 injection groups. There is no significant difference in body weight across each dosage group.male (n=5), female (n=5), total number is 10. **Table S3.** Comparison of clinical chemistry changes between the AAV-DX2 injection groups. There is no significant difference in all parameters of clinical chemistry across each dosage group.male (n=5), female (n=5), total number is 10. **Table S4.** Comparison of parameters of urinalysis changes between the AAV-DX2 injection groups. There is no significant difference in all parameters of urinalysis across each dosage group.male (n=5), female (n=5), total number is 10. **Fig S4.** Histopathology analysis in DRG region between AAV-DX2 injection groups. Hematoxylin and Eosin staining of DRG region. There is no significant difference in all parameters of urinalysis across each dosage group. **Fig S5.** Immunoblot assay of cell survival efficacy by AAV-DX2 in MPTP-treated mice. Substantia nigra region was used for analysis.

## Data Availability

All relevant data are available within the article and its supplementary information files, or available from the corresponding authors upon reasonable request.

## Abbreviations

AIMP2: Aminoacyl tRNA synthetase complex interacting multifunctional protein 2
ssAAV: Single strand adeno-associated virus
scAAV: Adeno-associated virus
MPTP: 1-Methyl-4-phenyl-1,2,3,6-tetrahydropyridine
PD: Parkinson’s disease
CNS: Central nervous system
SHIRPA: SmithKline Beecham, Harwell, Imperial College, Royal London Hospital; phenotype assessment,
GFP: Green fluorescent protein
miR: MicroRNA
miRNA: MicroRNA
NRTN: Neurturin
GDNF: Glial cell line-derived neurotrophic factor
AADC: Aromatic L-amino acid decarboxylase
