# Supplementary material for: Brain-targeted delivery of neuroprotective survival gene minimizing hematopoietic cell contamination: implications for Parkinson’s disease treatment
Source: J Transl Med. 2024 Jan 13;22:53. doi: 10.1186/s12967-023-04816-x (PMC10790275; doi:10.1186/s12967-023-04816-x)
Supplement: Supplementary file 1 — Additional file 1: Fig S1. Expression of AAV-DX2 in SH-SY5Y. SH-SY5Y cells were infected with AAV-GFP and AAV-DX2. Total RNA in transfected cells were analyzed by quantitative RT-PCR. Fig S2. Immunoblot assay of cell survival efficacy by AAV-DX2. SH-SY5Y cells were infected with AAV-DX2. SH-SY5Y cells were infected with AAV-GFP and AAV-DX2. Cleaved-caspase-8 and Cleaved-caspase-9 were detected. Fig S3. Comparison of cellular signaling changes of AAV-DX2 using Dot Blot assay. Table S1. Abbreviation of parameters of clinical chemistry. Table S2. Comparison of body weight changes between the AAV-DX2 injection groups. There is no significant difference in body weight across each dosage group.male (n=5), female (n=5), total number is 10. Table S3. Comparison of clinical chemistry changes between the AAV-DX2 injection groups. There is no significant difference in all parameters of clinical chemistry across each dosage group.male (n=5), female (n=5), total number is 10. Table S4. Comparison of parameters of urinalysis changes between the AAV-DX2 injection groups. There is no significant difference in all parameters of urinalysis across each dosage group.male (n=5), female (n=5), total number is 10. Fig S4. Histopathology analysis in DRG region between AAV-DX2 injection groups. Hematoxylin and Eosin staining of DRG region. There is no significant difference in all parameters of urinalysis across each dosage group. Fig S5. Immunoblot assay of cell survival efficacy by AAV-DX2 in MPTP-treated mice. Substantia nigra region was used for analysis. [file 12967_2023_4816_MOESM1_ESM.docx]

**Supplementary material**

**Brain-Targeted Delivery of Neuroprotective Survival Gene Minimizing Hematopoietic Cell Contamination: Implications for Parkinson's Disease Treatment**

**Supplementary Method**

**1. Blood collection**

Blood samples for both hematology and clinical chemistry tests were procured at necropsy from the posterior vena cava. The collected sample size was approximately 0.6 mL, which was further divided into two separate portions of roughly 0.3 mL each, intended for the subsequent hematology and clinical chemistry evaluations. The collection of blood samples was performed using sterile, disposable polypropylene syringes which were fitted with sterile, disposable 25 G needles. Animal subjects were anesthetized via inhalation of isoflurane. For the hematology analysis, approximately 0.3 mL of the collected blood was placed into polypropylene tubes that contained an anticoagulant solution. This solution comprised 0.03 mL of 1.5% EDTA-2K aqueous solution, and the tubes were pre-dried in an incubator prior to sample collection. For the clinical chemistry analysis, around 0.3 mL of the blood sample was transferred into polypropylene tubes that contained 1.5 μL of heparin sodium (10000 units/10 mL) to avoid blood clotting. Finally, blood samples intended for clinical chemistry analysis underwent centrifugation at 5000 x g for 10 minutes at 4°C to separate the plasma.

**2. Clinical chemistry**All animals that were necropsied were included, with the exception of those that expired during the course of the study. The plasma samples derived from these subjects were examined using the Clinical Analyzer Model 7180, a product of the Hitachi High-Technologies Corporation. Prior to commencing any measurements, these plasma samples were subjected to a 2-fold dilution process with distilled water.

Regarding the storage of these samples, they were preserved In a frozen state at or below -65°C from the point of their collection until the time they are needed for analysis. A reserve sample, a part of each of the original samples, were stored under the same stringent conditions. All parameters and analysis methods have been explicitly delineated in the table provided below.

Parameters:

| Parameter | Abbr. | Unit | Method |
| --- | --- | --- | --- |
| Aspartate aminotransferase | AST | U/L | JSCC method |
| Alanine aminotransferase | ALT | U/L | JSCC method |
| Alkaline phosphatase | ALP | U/L | IFCC method |
| Lactate dehydrogenase | LD | U/L | IFCC method |
| Creatine kinase | CK | U/L | JSCC method |
| Glucose | GLU | mg/dL | Enzymatic (Gluc-DH) |
| Total bilirubin | BIL | mg/dL | Enzymatic (BOD) |
| Urea nitrogen | UN | mg/dL | Enzymatic (urease-LEDH) |
| Creatinine | CRE | mg/dL | Enzymatic |
| Total cholesterol | CHO | mg/dL | Enzymatic (cholesterol oxidase) |
| Triglycerides | TG | mg/dL | Enzymatic (GK-GPO with free glycerol elimination) |
| Phospholipids | PL | mg/dL | Enzymatic (choline oxidase) |
| Inorganic phosphorus | IP | mg/dL | Enzymatic (maltose phosphorylase) |
| Calcium | CA | mg/dL | OCPC |
| Sodium | NA | mEq/L | Ion-selective electrode |
| Potassium | K | mEq/L | Ion-selective electrode |
| Chloride | CL | mEq/L | Ion-selective electrode |
| Total protein | TP | g/dL | Biuret |
| Albumin | ALB | g/dL | BCG |
| Albumin-globulin ratio | A/G | − | Calculated |
| JSCC: Japan Society of Clinical Chemistry  IFCC: International Federation of Clinical Chemistry and Laboratory Medicine | | | |

**3. Urine collection**

The urinalysis process occurred at three-week intervals after the initial dosage administration. The reagent strips that were used in this procedure were MULTISTIX®SG-L, supplied by Siemens Healthcare Diagnostics K.K. To collect urine samples, the subjects were swiftly relocated to metabolic cages, which marked the start of the urine collection process.

Urine samples, each approximately 0.1 mL in volume, were collected via syringes from urine that accumulated for a period of 4 hours since the initiation of the collection process. Once the division of the samples was complete and the urinalysis was conducted, any remaining urine and post-analysis samples were disposed of.

If the desired volume of urine was not collected within the 8-hour window, or any parameter could not be analyzed, the sampling process was allowed to continue for a maximum duration of 2 days from the following day.

**Supplementary Figures**

**Supplementary Fig. 1 Expression of AAV-DX2 in SH-SY5Y.** SH-SY5Y cells were infected with AAV-GFP and AAV-DX2. Total RNA in transfected cells were analyzed by quantitative RT-PCR.

**Supplementary Fig. 2 Immunoblot assay of cell survival efficacy by AAV-DX2**. SH-SY5Y cells were infected with AAV-GFP and AAV-DX2. Cleaved-caspase-8 and Cleaved-caspase-9 were detected.

**Supplementary Fig. 3 Comparison of cellular signaling changes of AAV-DX2 using Dot Blot assay.**

**Supplementary Table 1. Abbreviation of parameters of clinical chemistry.**

**Supplementary Table 2**. **Comparison of body weight changes between the AAV-DX2 injection groups.** There is no significant difference in body weight across each dosage group. male (n=5), female (n=5), total number is 10.

**Supplementary Table 3. Comparison of clinical chemistry changes between the AAV-DX2 injection groups.** There is no significant difference in all parameters of clinical chemistry across each dosage group. male (n=5), female (n=5), total number is 10.

**Supplementary Table 4. Comparison of parameters of urinalysis changes between the AAV-DX2 injection groups.** There is no significant difference in all parameters of urinalysis across each dosage group. male (n=5), female (n=5), total number is 10.

**Supplementary Fig. 4 Histopathology analysis in DRG region between AAV-DX2 injection groups.** Hematoxylin and Eosin staining of DRG region. There is no significant difference in all parameters of urinalysis across each dosage group.

**Supplementary Fig. 5 Immunoblot assay of cell survival efficacy by AAV-DX2 in MPTP-treated mice**. Substantia nigra region was used for analysis.

**Supplementary Fig 1**

**
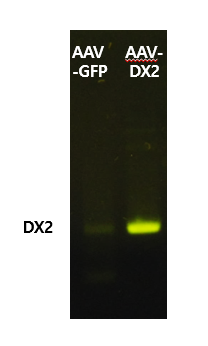
**

**Supplementary Fig 2**

**
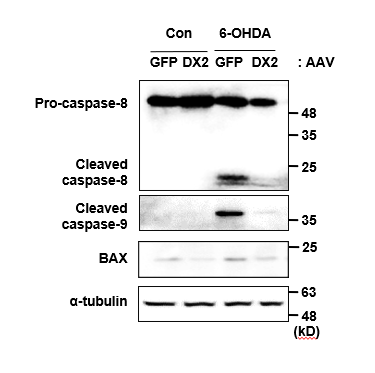
**

**Supplementary Fig 3**

**
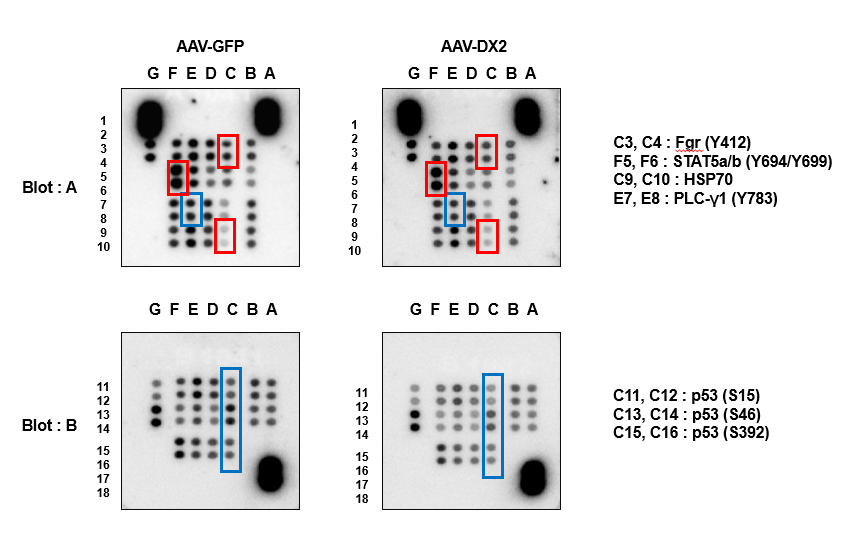
**

**Supplementary Table 1**


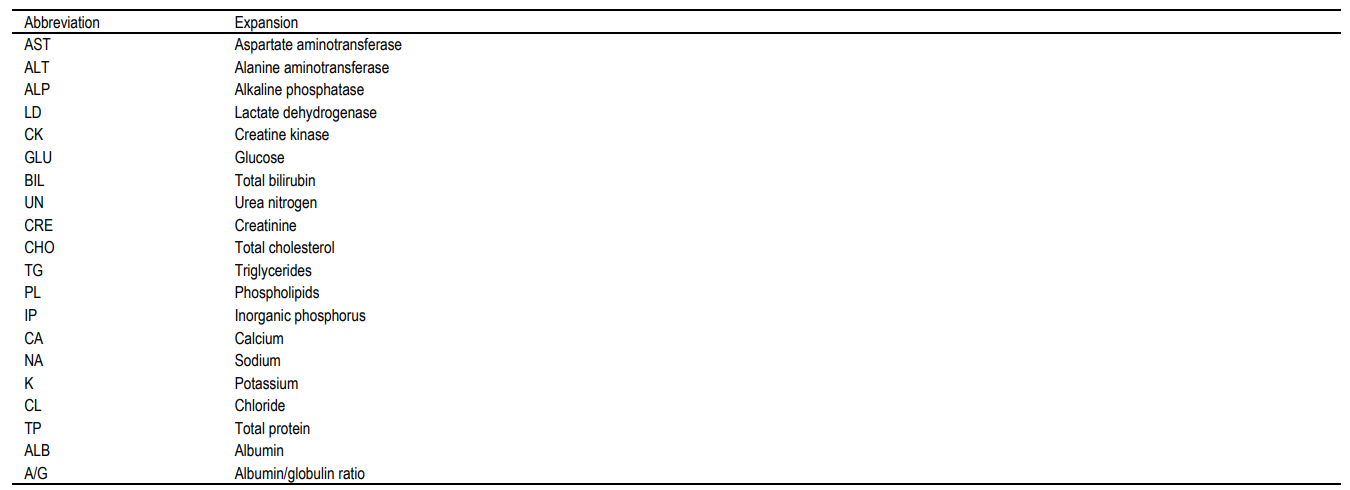

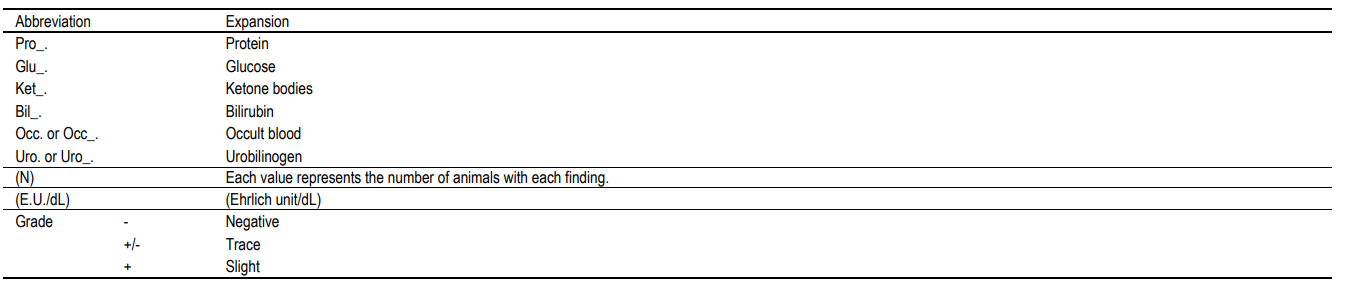


**Supplementart Table 2**


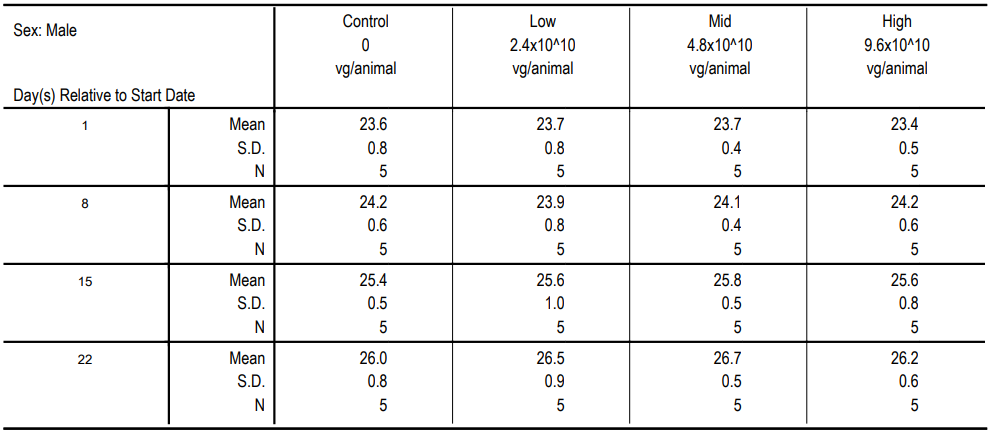

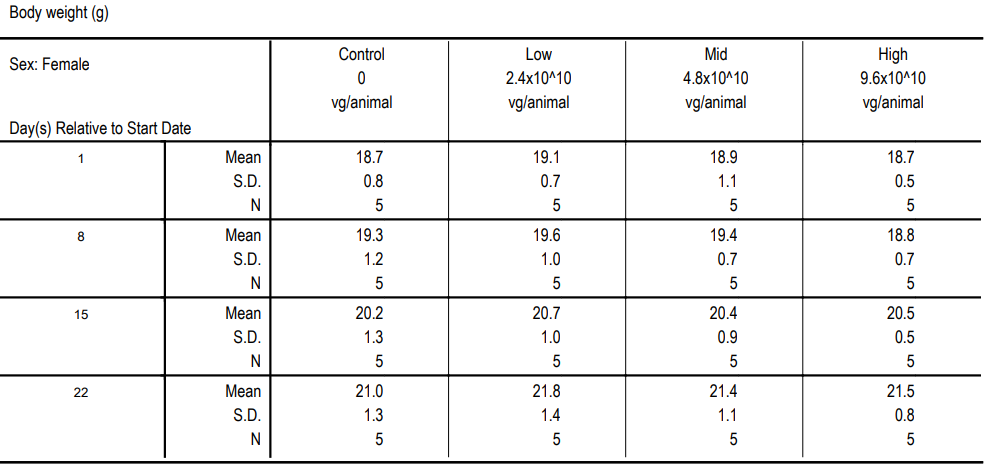


**Supplementary Table 3**


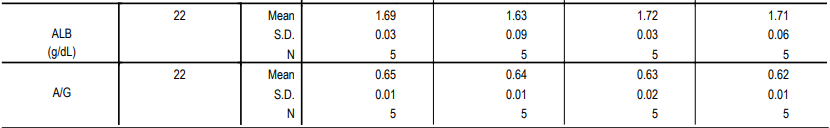

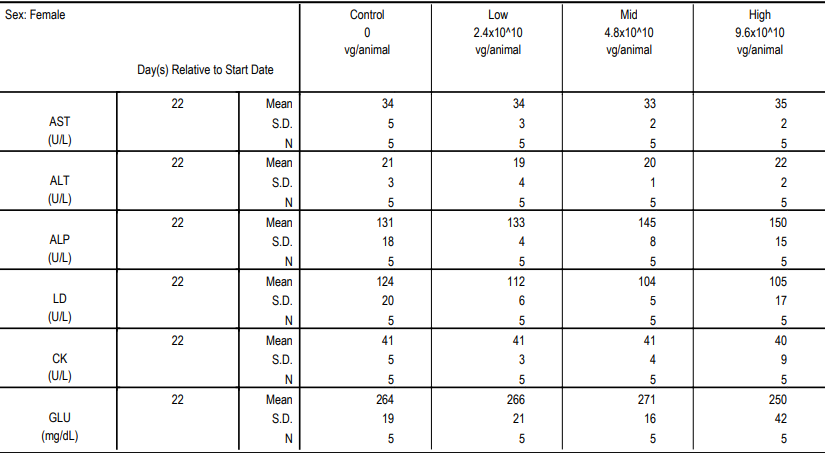

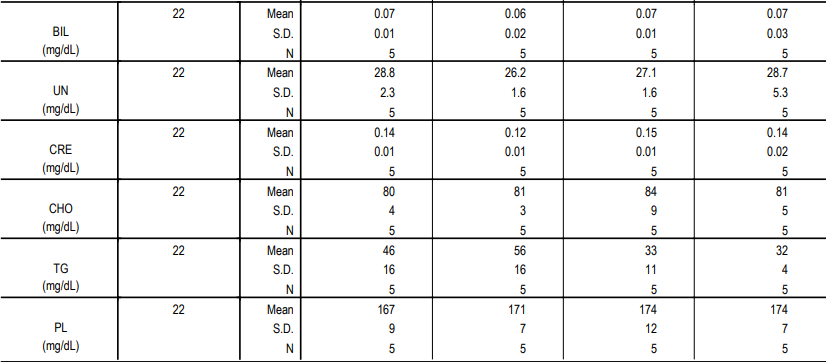

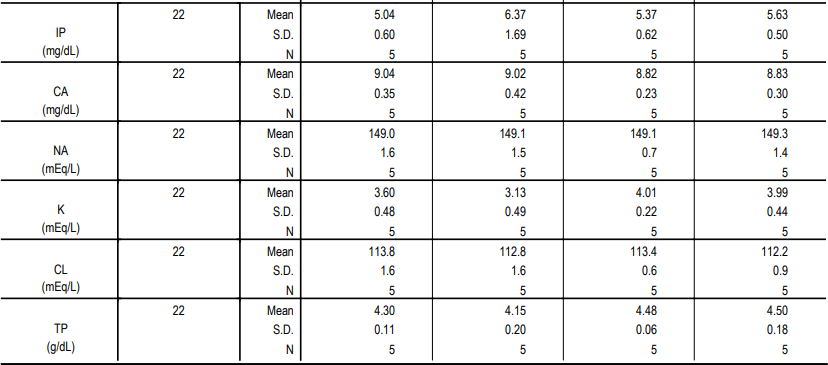


**Supplementart Table 3 (Continued)**


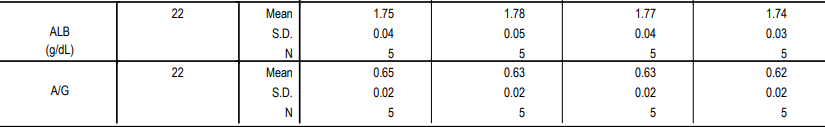

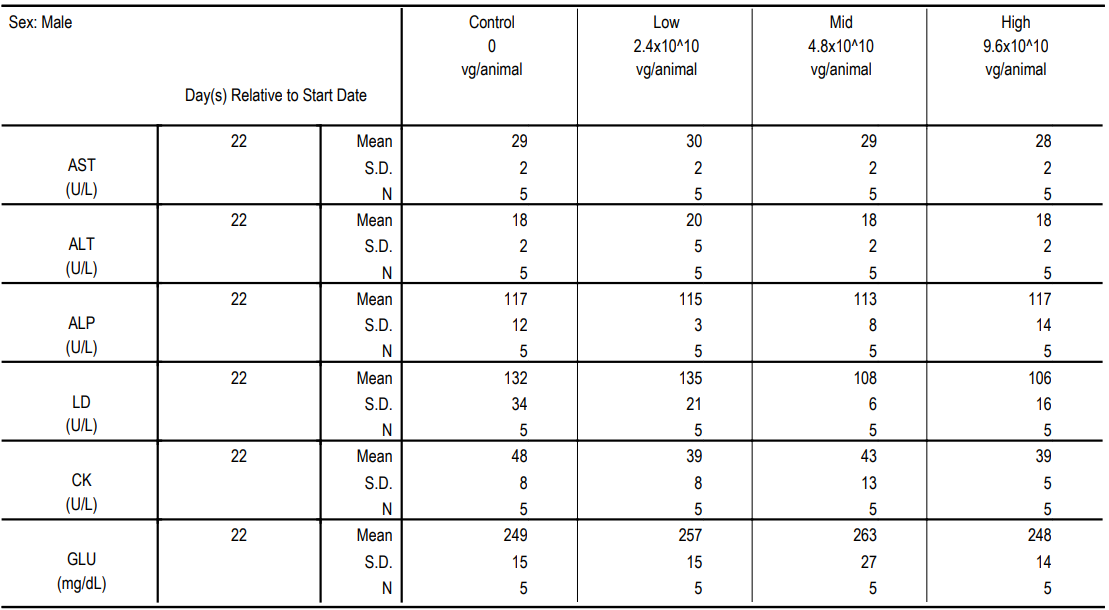

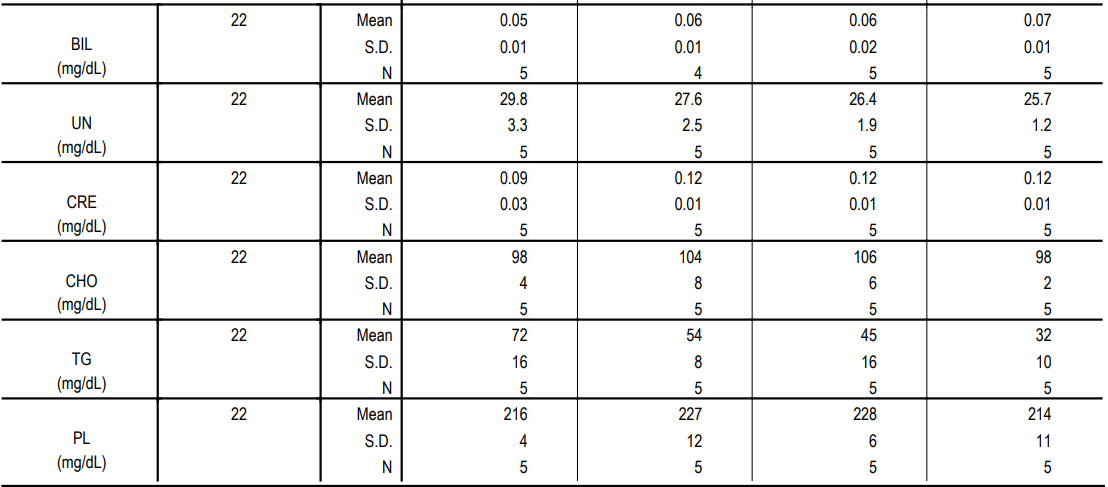

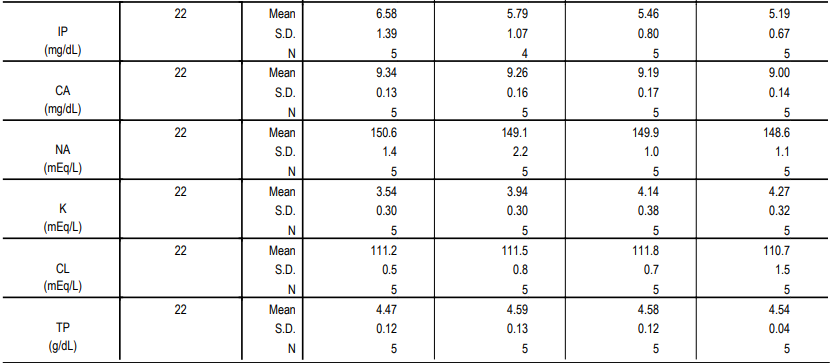


**
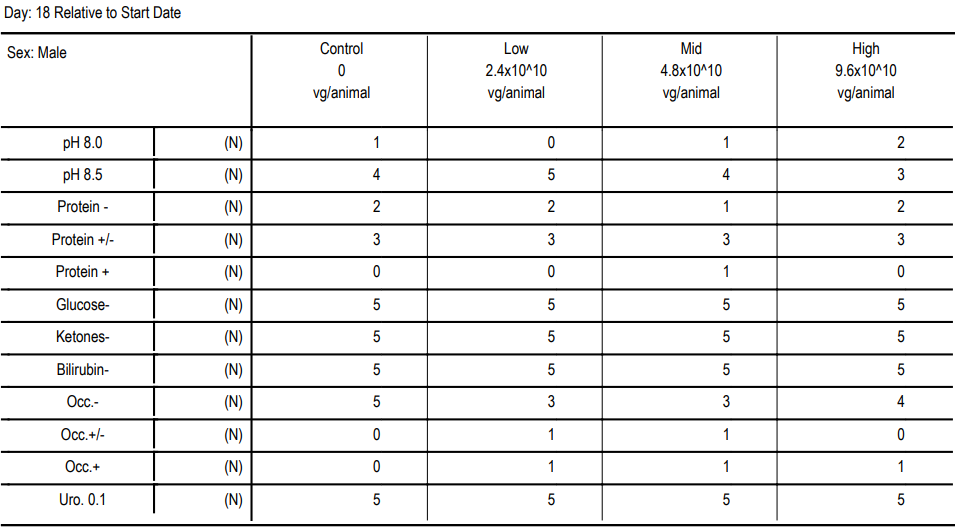
Supplementary Table 4**

**
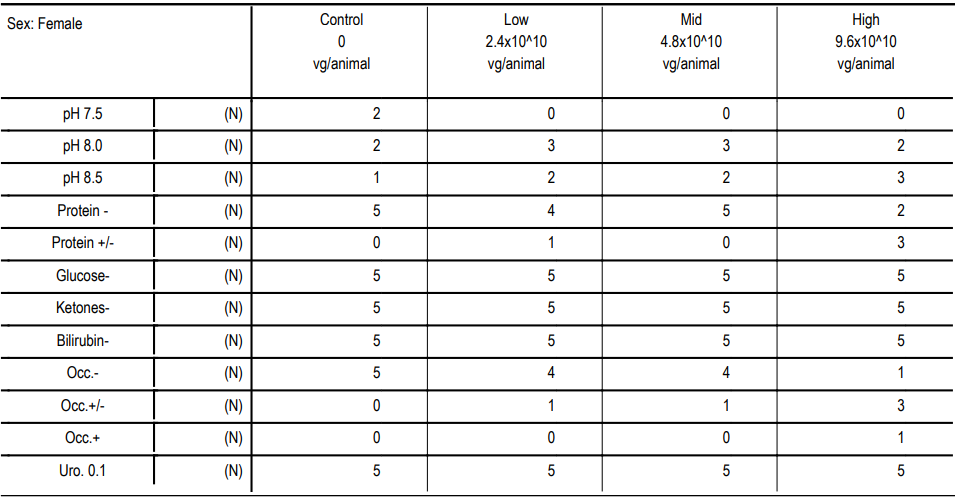
**

**Supplementary Fig 4**

**
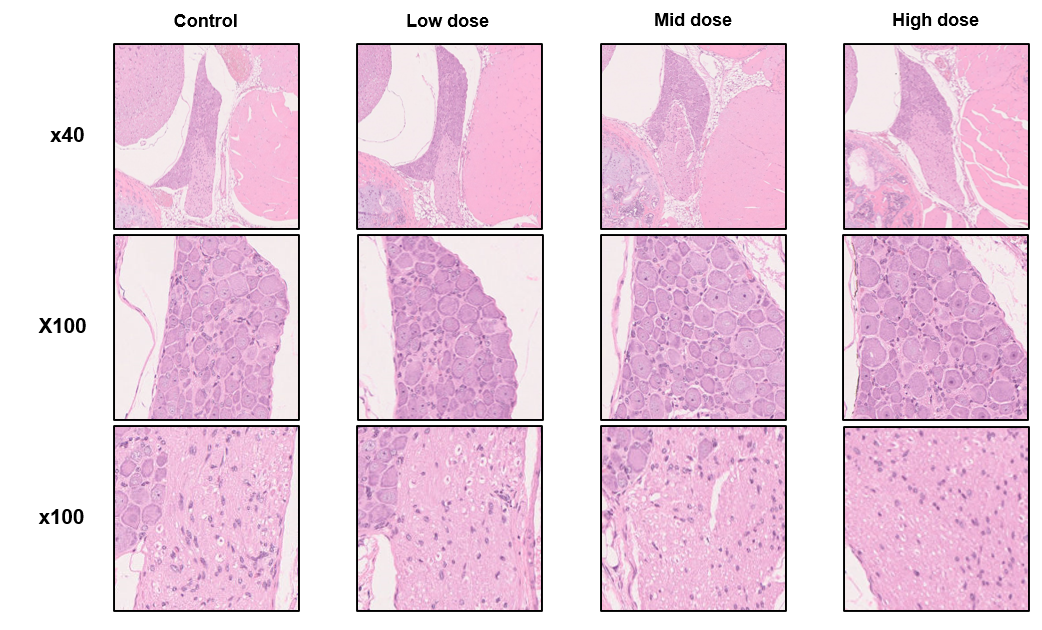
**

**Supplementary Fig 5**

**
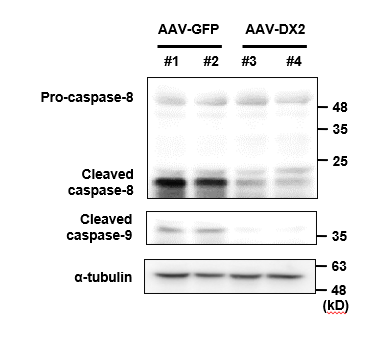
**
